# Gender moderates the association between chronic academic stress with top-down and bottom-up attention

**DOI:** 10.3758/s13414-022-02454-x

**Published:** 2022-02-17

**Authors:** Bradley J. Wright, Kira-Elise Wilson, Michael Kingsley, Paul Maruff, Jian Li, Johannes Siegrist, Ben Horan

**Affiliations:** 1 Department of Psychology and Counselling, La Trobe University, Bundoora, VIC Australia; 2 Holsworth Research Initiative, La Trobe University, Bendigo, VIC Australia; 3 Department of Exercise Sciences, University of Auckland, Auckland, New Zealand; 4 The Florey Institute of Neuroscience and Mental Health, University of Melbourne, Parkville, VIC Australia; 5 Department of Environmental Health Sciences, University of California, Los Angeles, Los Angeles, CA USA; 6 Centre for Health and Society, Henrich-Heine-University, Düsseldorf, Germany; 7 School of Engineering, Deakin University, Geelong, VIC Australia

**Keywords:** CONVIRT, Reaction time, Heart rate variability, Effort-reward imbalance, Anxiety

## Abstract

Research on the relationship between chronic stress and cognition is limited by a lack of concurrent measurement of state-anxiety, physiological arousal, and gender. For the first time, we assessed the impact of these factors on top-down/conscious (simple and choice reaction time) and bottom-up/reflexive (saccadic reaction time) measures of attention using CONVIRT virtual-reality cognitive tests. Participants (*N* = 163) completed measures of academic stress (effort-reward imbalance; ERI) and state-anxiety while heart-rate variability was recorded continuously throughout the experiment. Gender moderated the association between academic stress with the top-down measures (*b* = -0.002, *t* = -2.023, *p* = .045; *b* = -0.063, *t* = -3.080, *p* = .002) and higher academic stress was associated with poorer/slower reaction times only for male participants. For bottom-up attention, heart rate variability moderated the relationship between academic stress and saccadic reaction time (*b* = 0.092, *t* = 1.991, *p* = .048), and only female participants who were more stressed (i.e., ERI ≥ 1) and displayed stronger sympathetic dominance had slower reaction times. Our findings align with emerging evidence that chronic stress is related to hyperarousal in women and cognitive decrements in men. Our findings suggest that higher ERI and sympathetic dominance during cognitive testing was associated with poorer bottom-up attention in women, whereas for men, academic stress was related with poorer top-down attention irrespective of sympathovagal balance.

**Supplementary Information:**

The online version contains supplementary material available at 10.3758/s13414-022-02454-x.

## Introduction

Performance on standard neuropsychological tests may be impacted by state anxiety (Dorenkamp & Vik, 2018) and chronic stress (Kuhnell et al., 2020; Landolt et al., 2017). As these factors have been rarely considered concurrently, it is, however, unclear which factor is most influential. A systematic review of the impact of anxiety on cognitive performance suggests that the findings are inconsistent and potentially explained by several factors, including gender, physiological reactivity, chronic stress and, importantly, the type of cognitive task (Dorenkamp & Vik, 2018). Specifically, higher order tasks appear to be more affected by state anxiety than lower cognitive load tasks (Maloney et al., 2014). Conversely, the small amount of literature reporting the association of chronic stress with poorer cognition has focussed on simple attention tasks (Kuhnell et al., 2020; Landolt et al., 2017). In this paper we extend this small research and assess whether chronic stress is related to simple measures of top-down (selective) and bottom-up (reflexive) attention after considering the role of state mood, maladaptive coping, gender, and physiological arousal. Such knowledge is a necessary first step towards understanding if such factors impact performance on measures of attention that are often used to inform diagnosis and prognosis for a variety of neuropsychological conditions.

### Gender

Gender differences have been reported for measures of chronic stress, coping, state anxiety, physiological reactivity, and performance on attention tasks of simple and choice reaction time. Specifically, there exists a mixed literature that largely suggests women respond more slowly on measures of simple, choice, and ocular (i.e., saccadic) reaction times (Bargary et al., 2017; Der & Deary, 2006; Reimers & Maylor, 2006). Further, women report higher levels of maladaptive coping known as ‘overcommitment’ (Hodge et al., 2020), chronic stress (Matud, 2004), sympathetic nervous system dominance under stress (Koenig & Thayer, 2016; Pushpanathan et al., 2016), and state anxiety (Fernández-Castillo & Caurcel, 2015). Additionally, there is evidence of the differential gender effects of state anxiety on cognitive performance, with females being more vulnerable to the effects of anxiety than males (King et al., 1978; Sarason & Minard, 1962). There is also a larger literature that suggests that the effects of anxiety upon cognition are not gender-specific (Dorenkamp & Vik, 2018). It is unknown, however, if the relationship between chronic stress and measures of attention are moderated by gender.

### Chronic stress

Although substantially less studied than the effects of state anxiety (Sandi, 2013), the impact of chronic stress on cognition appears to be more homogenous, with chronic stress consistently associated with poorer cognitive performance. Despite substantial literature on the relationship between chronic stress, physiology, and cognition (see Wolf et al., 2016, for a review), most research has focussed on measures of memory function, with fewer considering the impact on attentional processes (Sandi, 2013). Further, of those that have considered tasks that primarily assess attentional processes, to the best of our knowledge only two studies (Kuhnell et al., 2020; Landolt et al., 2017) have incorporated a physiological measure of arousal in their design.

In a recent study, the association between effort-reward imbalance (ERI; Siegrist, 1996), a measure of chronic workplace stress, with visual processing speed and choice reaction time (CRT) was moderated by heart rate variability (Landolt et al., 2017). Specifically, this study used an acute stress test (cold-pressor Maastricht Acute Stress Test (MAST); Smeets et al., 2012) at two timepoints several months apart, and only during the ‘high-stress period’, associations between high ERI, high sympathetic dominance and poorer reaction time speeds (i.e., CRT) were evident. Similarly, research with university students showed that overcommitment, an intrinsic component of the ERI workplace model, characterised by an enduring pattern of poor coping and an inability to withdraw from work, was related to poor attention (i.e., CRT) among students who displayed sympathetic dominance as measured by the low-frequency to high-frequency (LF/HF) ratio of heart rate variability in the phase before the cognitive test (Kuhnell et al., 2020). Landolt et al. (2017) also considered the role of acute stress on cognitive performance, and suggested that acute stress had little impact, but that chronic workplace stress did. The contribution of autonomic arousal to the relationship between chronic stress and attention cannot be underestimated, with both studies reporting that heart rate variability moderated the relationship between measures of chronic stress and attention. However, differences in measures of attention that may arise due to more conscious (top-down) or reflexive (bottom-up) attention tasks were not included in the design of either of these studies.

### Physiological reactivity

It has been suggested that solely confining assessment to psychological stress or anxiety at testing may not be sufficient in determining if these factors are associated with poor cognitive performance (Dorenkamp & Vik, 2018). Similarly, assessing physiological reactivity in the absence of psychological data limits interpretation. The best approach may involve a concurrent consideration of both psychological and physiological responses to cognitive testing (Dorenkamp & Vik, 2018; Maloney et al., 2014).

Psychological stress is associated with heightened hypothalamic pituitary adrenal axis activity and has consistently been associated with decrements in memory (see Sauro et al., 2003, for a meta-analysis), but the impact of autonomic arousal on measures of cognition has received less study. Several studies of visual attention to emotional stimuli have shown that higher heart rate variability may be associated with more adaptive top-down and bottom-up responses (Park & Thayer, 2014), and recent research assessing the effects of chronic stress on top-down visual attention with simple reaction time (SRT) and CRT has shown that higher sympathetic and lower parasympathetic measures of heart rate variability at pre-test were related with *poorer* CRT (Kuhnell et al., 2020).

### State anxiety

Research on how state anxiety influences the performance of cognitive tasks has shown that both higher and lower levels of anxiety are related with poor performance, with other studies also reporting no association between anxiety and performance (Dorenkamp & Vik, 2018). The mixed findings may be explained by task complexity. State anxiety can improve performance on simple tasks but impair performance in higher-order tasks (Bolmont et al., 2000). Although mood states have received less empirical attention, fatigue, confusion, and hostility have been associated with poorer CRT and visual attention (Bolmont et al., 2000). Despite some study on the effects of state anxiety upon attention, to our knowledge, the potential differential effects of chronic stress, mood, and autonomic arousal on measures of top-down and bottom-up attention has not been investigated.

### The present study

In this study, we used a virtual-reality cognitive test, ‘CONVIRT’, which uses eye-tracking technology to assess saccadic speed, and a thumb press to measure SRT and CRT (Horan et al., 2020) to assess visual attention. CONVIRT has been shown to induce autonomic arousal and has been validated with university students (Amato et al., 2020; Horan et al., 2020), who are known to report high levels of chronic academic stress (Kuhnell et al., 2020; Wege et al., 2017). We assessed the relationship between academic stress with tasks that predominantly measure top-down (SRT, CRT) and bottom-up (saccadic reaction time) attention, after controlling for age, overcommitment, state anxiety, fatigue, and the LF/HF measure of sympathovagal balance. Further, we assessed which of these variables, when considered concurrently, was most strongly associated with the cognitive outcomes. Finally, we sought to identify whether gender or LF/HF moderated the association between academic stress and cognitive outcomes. We anticipated that both higher academic stress and sympathetic dominance would only be associated with poorer performance on the top-down tasks (SRT, CRT) and hypothesised that higher state anxiety and sympathetic dominance during testing would be associated with improved performance on the bottom-up measure of visual processing speed. While we anticipated that there would be gender differences in overcommitment, state anxiety, fatigue, and sympathovagal balance, we did not expect gender to moderate the association between chronic academic stress and the cognitive measures. Given the research to date, we predicted that higher sympathovagal balance would only moderate the association between chronic academic stress and top-down measures of attention.

## Method

### Participants

Australian university students (*N* = 163, 80 females) aged between 18 and 34 years (*M* = 22.77; *SD* = 3.40) were approached face-to-face and invited to participate in the study using a recruitment script. A power analysis using G*Power 3 (Faul et al., 2009) for a hierarchical regression with eight predictors with an expected high effect (*f* = .35; Kuhnell et al., 2020; Landolt et al., 2017;), and power set at .80, determined that an *N* of 52 was required to detect an association at *p =* .05. Participants were included if they could read English and were currently enrolled at an Australian university full-time. Participants were excluded if they self-reported that they were ill at the time of the study, had ongoing mental or physical health problems, considered themselves physically fragile, or had sustained a concussion within the 6 months prior to the study. Some of these exclusion criteria have been reported to confound heart rate variability assessment (Task Force of the European Society of Cardiology the North American Society of Pacing Electrophysiology, 1996), and others relate to adverse experiences in virtual-reality environments (Bouchard et al., 2009; Stanney et al., 2002). Prior to participation, and in line with institutional ethics approval (HEC S17-117), participants provided written informed consent, and all participants were compensated for their time with a double pass to the cinema.

### Measures

#### Academic stress

The student version of the effort-reward imbalance questionnaire (ERI-U; Wege et al., 2017) was administered to measure chronic academic stress. The ERI-U contains 14 items across three scales: ‘Effort’ (e.g., “My study load has become more and more demanding”); ‘Reward’ (e.g., “I receive the respect I deserve from my fellow students”); and ‘Overcommitment’ (e.g., “As soon as I get up in the morning, I start thinking about study problems”). Items are measured on a 4-point Likert scale, from ‘strongly disagree’ to ‘strongly agree’. An effort-reward ratio (ERI ratio) can be computed from the ‘effort’ and ‘reward’ items, where ERI ratio scores greater than 1 suggest higher stress and risk of illness (Siegrist & Li, 2016). An ERI ratio score and ERI group variable (dichotomised by those with ERI < 1, ERI > 1) were both calculated. The Cronbach’s alpha (α) in the present study were acceptable (Hair et al., 2010), Effort = .66, Reward = .63, Overcommitment = .76.

#### State anxiety

The short-form of the State-Trait Anxiety Inventory (STAI-6; Marteau & Bekker, 1992) comprises six items (e.g., “I feel upset”) prompting participants to rate their current anxiety level on a 4-point Likert scale, from ‘not at all’ to ‘very much so’. The STAI-6 items demonstrate very strong convergent validity with the full-form STAI, and strong internal consistency (present study = .80; Tluczek et al., 2009). Higher scores correspond to higher reports of state anxiety.

#### Fatigue

The ‘fatigue’ subscale from the Profile of Mood States (Shacham, 1983) comprises six items in total, in which participants are prompted to rate emotive words in accordance with their current mood state (e.g., “Lively”) on a 5-point Likert scale, from ‘not at all’ to ‘extremely’. Higher scores correspond with higher reports of state fatigue. Only the fatigue subscale from the Profile of Mood States was used in this study. The scale exhibited strong item reliability, Cronbach’s α = .86.

#### Heart rate variability

Heart rate variability data were calculated from recorded cardiac cycle durations (RR-intervals) obtained using a wireless Polar RS800CX heart rate monitor and chest belt (Polar, Finland), as an indirect measure of autonomic nervous system activity. The Polar RS800CX is a reliable instrument producing ECG-comparable measures of heart rate variability (Radespiel-Tröger et al., 2003; Vieira et al., 2012; Weippert et al., 2010). To calculate short-term heart rate variability in 5-min epochs, non-physiological RR-intervals were removed where beat-to-beat differences were identified to be more than 50% of resting values, and consecutive RR-intervals analysed using customised software (LabVIEW 2016; National Instruments, UK). A Hanning window and Fast Fourier Transformation of 1-min segments with 50% overlap was used to determine the power spectral density in LF (0.04–0.15 Hz) and HF (0.15–0.40 Hz) bandwidth, as previously described (Landolt et al., 2017). Mean LF/HF ratio was calculated for both baseline and CONVIRT phases of the study. The LF/HF ratio of spectral heart rate variability was computed, as it has previously been proposed to represent the ‘push-pull’ dynamic between the sympathetic and parasympathetic divisions of the autonomic nervous system (Lombardi et al., 1996), and a higher LF/HF ratio is associated with heightened activity of the sympathetic nervous system and elevated stress (Billman, 2013; Delaney & Brodie, 2000).

#### The CONVIRT battery

The CONVIRT battery is a computerised test battery that features a virtual reality (VR) test environment with embedded eye-tracking capabilities (Amato et al., 2020; Horan et al., 2020). It contains three tests of cognitive performance, measuring bottom-up attention (the saccadic reaction time test; SAC-VR), top-down attention via SRT (the detection test; DET-VR), and CRT (the identification test; IDN-VR). In each test, participants wear a VR head mounded display and view an animated virtual horserace from the first-person perspective of a jockey on horseback. The CONVIRT tool runs on a Gigabyte P35 laptop, and comprises the FOVE 0 Eye Tracking virtual-reality Headset, CONVIRT VR application, and a customised wireless riding crop with a push-button pressed through a thumb press by participants. Participants respond to stimuli by either fixating their eyes on a stimulus (SAC-VR) or by pushing the riding-crop push-button with the thumb press (DET-VR and IDN-VR). Prior to each trial, instructions appear on the virtual display, and participants perform a short practice test. The CONVIRT battery has high test-retest reliability (SAC-VR, *r* = .79; DET-VR, *r* = .90; IDN-VR, *r =* .88) and convergent validity with relevant clinical measures (Horan et al., 2020), and is sensitive to the cognitive changes elicited through oral alcohol consumption (Amato et al., 2020).

##### Visual processing speed (SAC-VR)

In this predominantly bottom-up attention test, participants need to fixate their gaze on a grey sphere in the center of their view. Green-colored eye symbols illustrate where each eye of the participant’s gaze resides in the environment. A blue sphere randomly appears in the virtual reality environment and the participant needs to move their gaze to it as quickly as possible. When the green eye symbols converge on the blue sphere, representing that the participant is looking at the object, the participant receives auditory and visual feedback of the sphere exploding. The grey circle then reappears in the environment and the process resets and continues. The SAC-VR assesses bottom-up attention as it only measures the time taken, or response latency, to initiate and begin the saccade (Orban de Xivry & Lefèvre, 2007). This is taken as the time for the participant’s gaze to reach 50% of the distance towards the blue sphere, and this movement occurs reflexively. The remaining 50% of the distance towards the target (i.e., blue sphere) includes portions of deceleration and accuracy adjustments involving other neural processes (Orban de Xivry & Lefèvre, 2007), and for this reason, and to separate the influence of top-down processes, are not considered in the SAC-VR measure.

##### Simple reaction time (DET-VR)

The DET-VR detection test predominantly measures top-down attention. Participants use the button on the riding crop to respond as quickly as possible to a yellow triangle randomly presented in the virtual reality environment. The triangle will then disappear for a duration of between 1 and 2.37 s, before an orange triangle appears at a random point on the 180° arc in front of the participant. This process repeats for a duration of 120 s and 35 triangles are presented in total. In line with the internationally used Cogstate SRT measure, used in both the Landolt et al. (2017) and Kuhnell et al. (2020) studies, SRT was measured based on the time elapsed (in milliseconds) between each triangle appearing and the thumb press of the button on the riding crop. False positives occur when the button is pressed but no triangle is present. Detection tests with false positives constituting more than 10% of all button presses are excluded. No tests were excluded based on this criterion.

##### Choice reaction time (IDN-VR)

In this identification test (IDN-VR) predominantly measuring top-down attention and simple decision-making, participants are presented with blue and orange triangles and spheres at random times and locations along an invisible 180° arc in front of them, but are instructed to only respond to the presentation of an orange sphere. The test is completed after 120 s, with 31 shapes presented in total. Choice reaction time is measured by the time elapsed (in milliseconds) between each orange sphere appearing and the riding crop button being pressed. False positives occur when the button is pressed prior to the presentation of the correct stimuli, or in response to the distractor stimuli. Identification tests with false positives constituting more than 20% of all button presses are excluded. No tests were excluded based on this criterion.

### Procedure

Participants provided informed consent upon arrival at the testing laboratory, before fitting the wireless heart rate chest belt in privacy. A viable heart rate signal was confirmed prior to beginning the experiment. The heart rate chest belt remained attached for the full duration of the experiment.

#### Baseline phase (M = 8.57 min, SD = 2.61 min, range = 5–24 min)

After successfully attaching the heart rate monitor, participants were seated in a quiet testing room and completed a questionnaire pack comprising a demographics survey, the ERI-U, the STAI-6, and the POMS ‘vigor’ and ‘fatigue’ scales. The baseline phase enabled the collection of baseline heart rate variability data, while minimising the potential impact of anticipatory arousal on cognitive performance – a phenomenon that we have previously observed in a similar study design (Kuhnell et al., 2020).

#### CONVIRT phase (M = 15.45 min, SD = 4.25 min, range = 11–25 min)

Upon completion of the questionnaire pack, participants sat alongside the Gigabyte P35 laptop, before being fitted with the FOVE 0 Eye Tracking virtual-reality Headset and wireless riding crop. The three CONVIRT tests were completed as described above, with participants receiving instructions on-screen and the opportunity to undertake a practice test before each trial.

### Data analysis

All analyses were performed using the IBM SPSS Statistics computer software package (Version 22.0). A series of paired two-tailed *t*-tests were used to assess if the heart rate variability indices differed between the baseline and the CONVIRT phases of the experiment. Independent two-tailed *t*-tests were used to assess gender differences on the continuous variables and a Chi-square analysis assessed if the proportion of students with scores greater than 1 were equivalent across genders. Bivariate Pearson correlation coefficients were calculated to investigate relationships between key variables. Three multiple moderated regressions were conducted to assess if gender, heart rate variability, or a combination of these measures moderated the association between ERI and performance on the CONVIRT measures of cognition (DET-VR, IDN-VR, and SAC-VR) after controlling for age, heart rate variability at baseline, overcommitment, state anxiety, and state fatigue. All statistical analyses were conducted using a criterion value of *p* < .05, and the moderation analysis was conducted using the SPSS PROCESS macro (Hayes, 2013) with bootstrapping (*N* = 5,000 resamples). All covariates that define products were mean centred within the PROCESS program and standardised variables (z-scores) were used to produce standardized Beta coefficients.

## Results

### Data management

The three CONVIRT and LF/HF measures were positively skewed (*p* < .01) and were corrected via logarithmic and square root transformations, respectively. No data were missing, and the assumptions for all parametric testing were satisfied. The transformed variables are used in all parametric tests.

### Physiological changes across CONVIRT phases

As anticipated, the LF/HF measures were lower in the CONVIRT phase compared with pre-test, reflecting a reduction in sympathetic dominance for the entire sample, *t*_162_ = 3.65, *p* < .001, *d* = 0.29. There were no gender differences in changes in sympathovagal balance across phases *F*_1,161_ = 0.25, *p* = .618, *d* = .08.

### Normative and gender comparisons

The mean ERI of the student sample (*M =* 0.85; *SD* = 0.26) was lower (*p* < .001, Cohen’s *d* = 0.79) than data from a medical education sample (*N* = 406, *M* = 1.09, *SD* = 0.32; Wege et al., 2017; *N* = 200, *M* = 0.99, *SD* = 0.21; Hahn et al., 2017), and lower (*p* < .001, Cohen’s *d* = 0.49; *p* < .001, Cohen’s *d* = 0.52, *p* < .001, Cohen’s *d* = 0.89) than German and Italian university students (*N* = 689, *M* =1.00, *SD* = 0.32; Hilger-Kolb et al., 2018; males: *N* = 1,174, *M* = 1.10, *SD* = 0.50, females: *N* = 3,586, *M* = 1.20; *SD* =0.40, Porru et al., 2021) suggesting that participants in the present study were experiencing lower stress than many. The mean state anxiety score (*M* = 11.98; *SD* = 3.93) did not differ (*p* = .385, Cohen’s *d* = 0.10) from a university student sample just prior to an academic assessment worth 20% of their final grade (*N* = 122; *M =* 12.96; *SD* = 4.26; Taylor & Deane, 2002). Compared to men, women presented with higher overcommitment and lower LF/HF at both phases but did not differ in chronic academic stress (ERI), fatigue, anxiety, or any of the cognitive measures (Table 1).

**Table 1:** An assessment of gender differences on the key variables

	Male (*n* = 83) mean (SD)	Female (*n* = 80) mean (SD)	Cohen *d*	*p*
ERI	.81 (.25)	.88 (.28)	0.26	.134
ERI groups: *n*>1/*n*<1	26/83	27/80	0.03^a^	.868^a^
LF/HF baseline	4.73 (3.70)	3.67 (2.49)	0.36	.034
LF/HF CONVIRT	4.14 (3.29)	2.89 (2.18)	0.45	.005
Overcommitment	10.75 (3.33)	12.25 (3.44)	0.44	.005
Fatigue	12.72 (4.89)	12.39 (4.48)	0.07	.649
Anxiety	11.77 (4.26)	12.20 (3.57)	0.11	.488
SAC-VR	2.43 (0.07)	2.43 (0.10)	0.10	.134
DET-VR	2.44 (0.06)	2.44 (0.05)	0.01	.634
IDN-VR	2.55 (0.06)	2.56 (0.06)	0.17	.191

^a^Chi-square analysis, phi effect size computed, untransformed data for the SAC-VR, DET-VR, IDN-VR, LF/HF baseline, LF/HF CONVIRT are provided in the Table, transformed versions were used in all parametric tests

A comparison of the bivariate correlations between the key variables across gender reveals associations of the stress variables (i.e., ERI group and overcommitment) with the top-down and bottom-up attention scores (Table 2). Males with ERI scores > 1 had slower IDN-VR scores (simple reaction time). Males with higher overcommitment had slower SAC-VR and DET-VR scores, whereas women with higher overcommitment had lower, therefore better, SAC-VR performance than men, *z* = 2.79, *p* = .003. Many of the other associations were similar between genders, but key exceptions were the positive anxiety with ERI group relationship for men, and the positive fatigue with ERI group association for women. The ERI group variable appeared to be a more sensitive predictor of the three attention variables than the ERI ratio score, with stronger associations on five of the six associations across gender. Of these six comparisons between ERI and ERI group, the difference was most stark for the IDN-VR variable with the magnitude of the difference significant for females *z* = 1.66, *p* = .048. Although using the ERI as a continuous variable may have improved power to detect relationships between variables, we chose to dichotomise the ERI variable at 1, based on the correlations and the expectation of increased health risk to participants with an ERI > 1 (Siegrist & Li, 2016). The criterion validity of this cut-off has been demonstrated in both experimental (e.g., Almadi et al., 2013; Landolt et al., 2017; Penz et al., 2019; Vrijkotte et al., 2000) and large epidemiological studies (e.g., Beschoner, et al., 2021; Dragano et al., 2017; Duchaine et al., 2020; Kivimäki et al., 2018) as a valid predictor of disease risk Additionally, the continuous ERI variable had clusters of outlying scores at either end of the distribution that were removed during scanning for multivariate outliers that may have led to an underestimate of the association of the predictor with the outcomes. As such, the ERI group variable was used in all subsequent regression models. As recommended (Montano et al., 2016), the continuous ERI variable was used in follow-up regressions with the aim of adding to the existing literature that has assessed the merits of the categorical and continuous measure of ERI. The findings from the continuous ERI variable are not disscussed in the present paper for the reasons outlined above, but the basic results are reported in Online Supplementary Material (OSM) Tables 1 and 2.

**Table 2:** Associations between the key variables, sorted by gender

		Age	1	2	3	4	5	6	7	8	9
1.OC	M	-.040									
	F	.015									
2.ERI	M	-.040	.525**								
	F	.064	.482**								
3.ERI group	M	-.080	.407**	.731**							
	F	.223*	.249*	.746**							
4.Fatigue	M	-.100	.229*	.182	.145						
	F	-.192	.249*	.278*	.128						
5.Anxiety	M	-.090	.386**	.333**	.308**	.444**					
	F	-.090	.227*	.309**	.116	.571**					
6.LF/HF baseline	M	-.030	.072	.131	.110	-.034	.012				
	F	.075	.184	.114	.047	.116	-.017				
7.LF/HF CONVIRT	M	.173	.066	.132	.126	.023	.048	.727**			
	F	-.046	.153	.031	-.024	.051	-.090	.534**			
8.SAC-VR	M	-.127	.218*	.086	.145	.103	.032	.001	-.031		
	F	-.070	-.220*	-.157	-.079	-.007	-.033	-.099	-.220		
9.DET-VR	M	.088	.217*	.158	.209	.079	.123	-.154	-.091	.274*	
	F	-.129	.015	.002	-.097	.204	.158	.089	-.101	.183	
10.IDN-VR	M	.101	.103	.190	.299**	-.107	-.096	-.129	-.057	.177	.719**
	F	-.111	-.055	-.042	-.176	.206	.215	.006	-.162	.237*	.757**

**Fig. 1:**
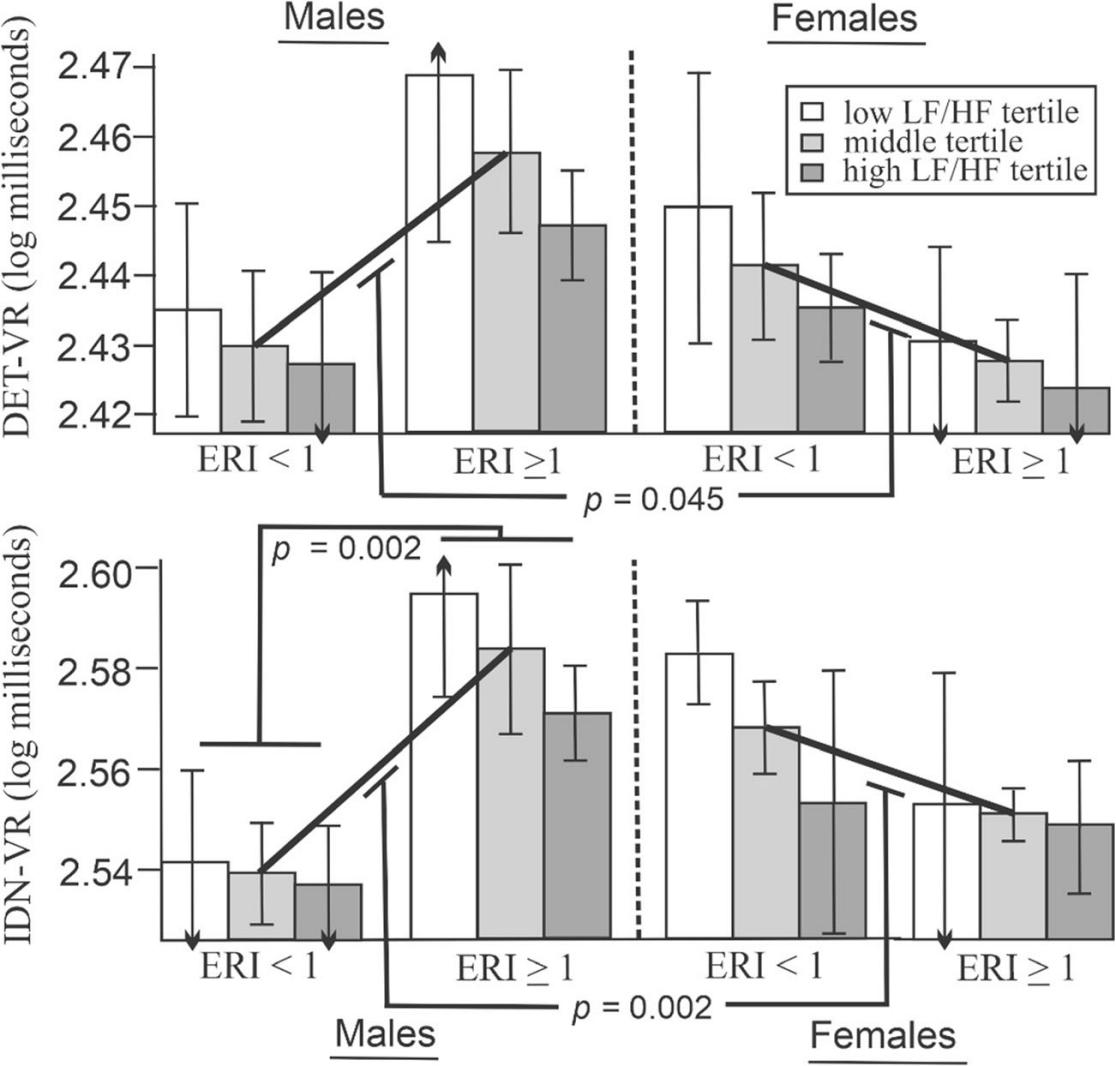
Gender, but not LF/HF differences in the relationship between ERI with attention (DET-VR) and choice-reaction time (IDN-VR). *Note.* Diagonal lines represent the direction of association between ERI group and DET-VR and IN-VR. Error bars depict standard error of the mean

The findings for the measures of simple reaction and choice reaction time were similar to gender differences noted in the direction of association between ERI group and cognitive performance for the DET-VR test, *b* = -0.002, *t* = -2.023, *p* = .045 and the IDN-VR test, *b* = -0.063, *t* = -3.080 *p* = .002 (Table 3). Neither heart rate variability nor the gender × heart rate variability tests of interaction for the ERI groups with DET-VR or IDN-VR measures were significant (Table 3). For males, ERI group and DET-VR were not related, *b* = 0.023, *p* = .174 whereas ERI and IDN-VR were positively related *b* = 0.048, *p* = .002. For females, the association of ERI group with DET-VR *b* = -0.012, *p* = .335, and IDN-VR *b* = -0.024, *p* = .074, were non-significant (Fig. 1). The opposing directions of association between genders may explain why the total model for both IDN-VR and DET-VR were non-significant.

**Table 3:** Assessing the direct and interactive effects of academic stress (ERI), gender and heart rate variability on performance of simple (DET-VR) and choice reaction time (IDN-VR)

Predictors	DET-VR				IDN-VR			
	*β*	se	*t*	*p*	*β*	se	*t*	*p*
ERI group	0.053	0.087	0.608	.597	0.103	0.088	1.117	.244
Gender	-0.015	0.083	-0.179	.858	0.066	0.084	0.785	.433
LF/HF CONVIRT	-0.119	0.106	-1.122	.264	-0.140	0.107	-1.308	.193
Age	0.041	0.081	0.504	.615	0.040	0.081	0.487	.627
Overcommitment	0.083	0.089	0.932	.353	-0.006	0.090	-0.072	.942
Anxiety	0.044	0.094	0.466	.642	-0.030	0.094	-0.319	.750
Fatigue	0.095	0.095	1.015	.312	0.063	0.094	0.675	.501
LF/HF baseline	-0.013	0.106	-0.122	.903	-0.022	0.094	-0.210	.834
ERI group × LF/HF	-0.015	0.081	-0.186	.858	0.015	0.081	0.183	.855
ERI group × Gender	-0.168	0.083	-2.023	.045	-0.257	0.083	-3.080	.002
ERI group × LF/HF × Gender	0.046	0.081	0.565	.573	0.075	0.082	0.923	.358

#### *Note*. DET

-VR total model *R*^*2*^= .07, *F*_11,151_ = 0.976, *p* = .475; IDN-VR total model, *R*^*2*^= .10, *F*_11,151_ = 1.356*, p* =.193; ERI group: 1= <1, 2 = < 1, Gender; 1= male, 2 = female. All covariates that define products were mean centred within the PROCESS program

**Fig. 2:**
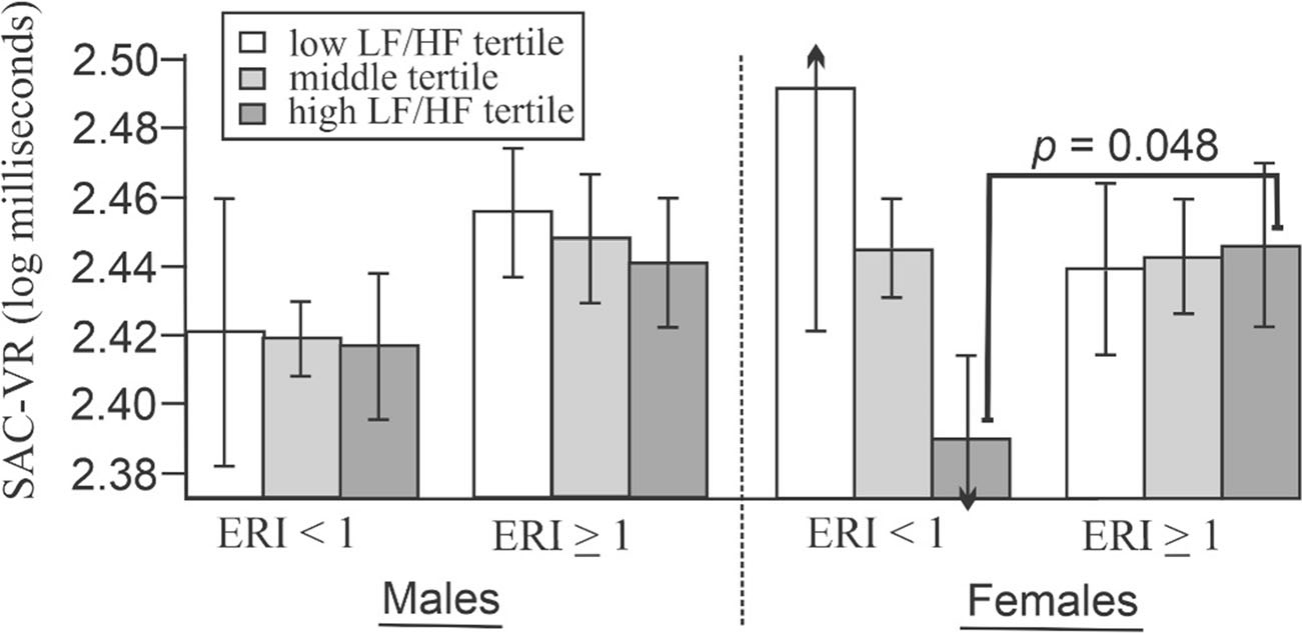
LF/HF moderates the association between ERI and visual processing speed (SAC-VR) in women, but not men. *Note.* Error bars depict standard error of the mean

Neither gender nor heart rate variability moderated the association between ERI group and SAC-VR, but the two factors interacted to explain the SAC-VR scores, *b* = 0.092, *t* = 1.991, *p* = .048 (Table 4). For males, the association between ERI group and SAC-VR was not moderated by heart rate variability , *b* = -0.009, *p* = .707, whereas for females the association was moderated by heart rate variability , *b* = 0.092, *p* = .039 (Fig. 2). The findings reveal that for females with higher ERI (i.e., > 1), higher LF/HF (sympathetic dominance) was related to slower SAC-VR performance, *b* = -0.054, *p* = .048, while lower LF/HF, *b* = 0.052, *p* = .139 and average LF/HF, *b* = -0.001, *p* = .965 were not related to SAC-VR scores.

**Table 4:** Assessing the direct and interactive effects of academic stress (ERI), gender, and heart rate variability on performance on saccadic reaction time (SAC-VR)

	SAC-VR			
Predictors	*β*	*se*	*t*	*p*
ERI group	0.075	0.087	0.865	.388
Gender	0.086	0.083	1.037	.301
LF/HF CONVIRT	-0.202	0.105	-1.919	.057
Age	-0.066	0.080	-0.820	.414
Overcommitment	-0.052	0.088	-0.590	.556
Anxiety	-0.067	0.093	-0.716	.475
Fatigue	0.113	0.092	1.218	.225
LF/HF baseline	0.014	0.105	0.130	.897
ERI group x LF/HF	0.122	0.080	1.516	.132
ERI group x Gender	-0.078	0.082	-0.946	.346
ERI group x LF/HF x Gender	0.161	0.081	1.991	.048

#### *Note.* SAC

-VR total model, *R*^*2*^ = .10, *F*_*11,151*_ = 1.45, *p* = .150; ERI group: 1= <1, 2 = < 1, Gender; 1= male, 2 = female. All covariates that define products were mean centred within the PROCESS program

## Discussion

In this study, academic stress had a greater impact on top-down attention in males than in females. That is, males with high chronic academic stress performed more poorly/slowly on the top-down attention tasks than females. In contrast, academic stress was not related to poor top-down attention for women, but on the bottom-up task that assessed saccadic reaction time, heart rate variability moderated the relationship between ERI and SAC-VR for women only. Specifically, females displaying higher ERI and sympathetic dominance had slower SAC-VR times than others. State anxiety was not associated with performance on any of the measures of attention.

### Gender

We predicted that despite expected differences on selected psychological and heart rate variability variables, gender would not moderate the asociation between chronic stress and cognition. This was not the case, with gender differences observed in the relationship of ERI with each of the cognitive tests.

Potentially, differences in coping (overcommitment) with academic stress (ERI) may provide some explanation for the observed gender differences. Specifically, despite similar levels of ERI, women had higher overcommitment and greater sympathetic dominance at pre-test and during CONVIRT, but did not differ from men in terms of changes between phases. Interestingly, there was no difference in state anxiety, and it is possible that the enduring pattern of maladaptive coping associated with overcommitment may go some way to explaining the disparate impact of chronic stress on attention between genders. The bivariate associations would support this premise, with higher overcommitment related to slower SAC-VR for men, and faster SAC-VR performance for women. Although speculative, it could be that the gender differences result from higher levels of enduring stress in women which are then pushed beyond a ‘tipping point’ when they do the CONVIRT tests. Specifically, the CONVIRT tests induce physiological arousal at a magnitude similar to a moderate acute stressor (Horan et al., 2020). This would align with one study *(N* = 39 males) that concurrently assessed chronic and acute stress with measures of psychological and physiological arousal, and found that subjects with high but not low chronic stress showed poorer higher-order decision-making in response to an acute stress test (Radenbach et al., 2015). Similar findings, whereby high ERI was only associated with poor simple decision-making (CRT) when LF/HF was low, are best understood by considering that higher LF/HF was related with poorer CRT irrespective of ERI (Landolt et al., 2017). In the present study, higher LF/HF among women with high ERI resulted in poorer SAC-VR performance. This result was only observed in highly stressed women on the bottom-up measure, and may be attributed to a more sensitive bottom-up measure, combined with a highly stressed subsample. There exists some underlying biological mechanisms that could support this assertion. There is evidence to suggest that women are more vulnerable than men to stress-induced hyperarousal in both healthy and clinical depression samples (Bangasser et al., 2018). Additionally, chronic stress can lead to volume reductions in prefrontal regions and the hippocampus, and volume increases in the amygdala that can render the individual more sensitive to the effects of acute stress (Lupien et al., 2009; Tse et al., 2014).

### Sympathovagal balance

With the exception of women on the SAC-VR test, LF/HF did not moderate the association between ERI and any of the other cognitive tests. A combination of high ERI and high LF/HF was a strong predictor of slower SAC-VR among women. This was not due to gender differences in LF/HF as the CONVIRT testing elicited an equivalent heart rate variability response across genders.

For the IDN-VR and DET-VR tests, heart rate variability during CONVIRT testing, after controlling for pre-testheart rate variability, was not associated with cognitive performance. The limited prior research has shown similar trends with cognitive testing associated with lower LF/HF from pre-test to cognitive testing phases (Kuhnell et al., 2020; Landolt et al., 2017). The pattern of association from previous studies also aligns with the trends observed in the current investigation with higher LF/HF (sympathetic dominance) associated with poorer cognition for men on all tests and women on the SAC-VR test. We do note, however, that previous research was from male-only (Kuhnell et al., 2020; Radenbach et al., 2015) or predominantly male samples (Landolt et al., 2017).

### Top-down and bottom-up attention

A review of the literature suggests differing brain regions of the parietal cortex are involved in top-down (superior parietal obule) and bottom-up attention (temporo-parietal junction) (Shomstein, 2012). Gender differences on the effect of chronic stress on tests was more pronounced in the top-down measures that involve the superior parital obule. Specifically, an ERI group with LF/HF interaction was only noted within the female gender on the bottom-up SAC-VR test, whereas the direction of association between ERI group and top-down attention (DET-VR, IDN-VR) differed by gender, and further, unlike females, males in the high ERI group performed more poorly on the IDN-VR test than males in the low ERI group.

Although simple and choice reaction time tasks have long been considered measures of top-down attention (Noudoost et al., 2010), the measure of visual processing speed (SAC-VR) in the present study differs from the Posner cueing task that has been used to measure both top-down and bottom-up attention (Posner, 1980). In the Posner cueing task, participants are provided with a cue (that is correct 80% of the time) to the direction of an impending stimulus at one of two points, whereas the SAC-VR test does not encourage covert shifts of attention to improve response latency. Additionally, unlike the Posner cuing task, the scene in the CONVIRT virtual-reality environment is dynamic and the stimuli appear randomly anywhere on the 360° arc.

Unconcious memory (e.g., Hollingworth, Matsukura, Luck, 2013; Ramey, Yonelinas, Henderson, 2019) or meaning-based guidance (Henderson & Hayes, 2017; Schütt et al., 2019) can influence the speed of a first saccade. The SAC-VR test, however, is less vulnerable to the potential influence of these top-down processes given the random appearance of the stimuli after the eyes have been fixated to the centre of the visual field (grey circle) for 2 s. Additionally, as only the first 50% of the movemnt towards the target is captured, the influence of other neural processes involved in deceleration and accuracy (Orban de Xivry & Lefèvre, 2007) are not considered within the SAC-VR measure. Finally, although visual searching of scenes is strongly influenced by top-down processes, the first saccade is driven by bottom-up control before transitioning to top-down control (see Schütt et al., 2019, for a review). Considered collectively, the SAC-VR test is likely a valid measure of bottom-up attention, so the gender differences on the tests could be due to differences in visual attention behaviour or top-down and bottom-up processes. Some research suports this notion with gender differences noted in fixaton points on faces (Coutrot et al., 2016; Shen & Itti, 2012) and use of bottom-up (males) or top-down (females) processes in visual reasoning tasks (Semrud-Clikeman et al., 2012). Further research is required however, to better test these assertions.

### Limitations and strengths

The findings from this study are based on a sample size of more than double that of similar studies and our design, which included a variety of psychological, biological, and behavioural responses in an immersive virtual-reality paradigm, is a first. However, as this appears to be the only research that has concurrently assessed chronic stress and state anxiety with markers of autonomic arousal and accounted for top-down and bottom-up attentional processes, we are cautious in making firm conclusions. The use of a categorical ERI variable is common in the literature given the proposed theoretical cut-point of ‘imbalance’ (Siegrist, 2016) and was appropriate for this low-stress sample that contained outliers at both ends of the distribution that may have led to spurious findings. However, we acknowledge that categorical variables can also lead to spurious outcomes (MacCallum et al., 2002).

Unlike most other research, our measures of cognition did not include components of higher-order cognition related with decision-making (Starcke & Brand, 2016) or memory performance (Sandi, 2013). This was a conscious design decision to focus on graduated complexity for the attention tasks, ahead of including higher-order executive function measures. Although the immersive CONVIRT experience provides a multitude of distracters (audio and moving visual environments) that increase physiological arousal more than conventional neuropsychological tests (Horan et al., 2020), the tasks within CONVIRT may produce a smaller cognitive load for participants than higher-order cognitive tests, and this is an important difference as higher cognitive loads are associated with increased sympathetic activity (Jerčić, Sennersten, & Lindley, 2020; Muthukrishnan, Gurja, & Sharma, 2017). Further, although we had heart rate variability measures at two timepoints, we only measured the other independent variables and cognitive outcomes once, and, as such, cannot infer causality between associations. To that end, within-group prospective comparisons provide a more sophisticated answer to these questions and the use of top-down and bottom-up attentional tasks within CONVIRT are useful in this regard as, unlike higher-order cognitive tasks, they are not confounded by practice effects. Finally, as our findings emanate from a sample of low-stressed university students, they may not generalise to other student populations or the general workforce. Specifically, the lower levels of chronic academic stress in this sample may explain why unlike in similar studies (i.e., Kuhnell et al., 2020; Landolt et al., 2017), sympathovagal balance did not interact with chronic academic stress to explain poor attention performance for the entire sample.

## Conclusion

Unlike in previous research, this study concurrently assessed the impact of chronic academic stress and state anxiety, including physiological arousal on both top-down and bottom-up measures of attention. Gender moderated the relationship between chronic academic stress and poor attention, with higher stress associated with poorer attention in males but not females. Moving forward, research is required to build on the small evidence showing an interaction between chronic stress and autonomic imbalance with attention. Further, our findings suggest that the effects of gender in this relationship warrant further investigation. Our findings align with emerging evidence that chronic stress is associated with hyperarousal in women and cognitive decrements in men (Bangasser et al., 2018). Potentially, our findings suggest that poorer bottom-up attention in women is more likely for those who show higher ERI and sympathetic dominance during cognitive testing. Whereas for men in this low-stress sample, chronic academic stress is associated with poorer attention irrespective of sympathovagal balance.

## Supplementary Information

ESM 1 (DOCX 38 kb)
