# Supplementary material for: Gender moderates the association between chronic academic stress with top-down and bottom-up attention
Source: Atten Percept Psychophys. 2022 Feb 17;84(2):383–95. doi: 10.3758/s13414-022-02454-x (PMC8888365; doi:10.3758/s13414-022-02454-x)
Supplement: Supplementary file 1 — (DOCX 38 kb) [file 13414_2022_2454_MOESM1_ESM.docx]

**Supplementary Tables.**

Supplementary Table 1. Assessing the direct and interactive effects of academic stress (ERI ratio), gender and heart rate variability on performance of simple (DET-VR) and choice reaction time (IDN-VR).

|  | DET-VR | | | | | IDN-VR | | | |  |
| --- | --- | --- | --- | --- | --- | --- | --- | --- | --- | --- |
| Predictors | | *β* | se | *t* | *p* | | *β* | se | *t* | *p* |
| ERI ratio (continuous) | | 0.050 | 0.074 | 0.561 | .576 | | 0.198 | 0.094 | 2.106 | .037 |
| Gender | | 0.036 | 0.076 | 0.470 | .858 | | 0.113 | 0.077 | 1.459 | .147 |
| LF/HF CONVIRT | | -0.081 | 0.076 | -0.761 | .448 | | -0.074 | 0.107 | 0.689 | .492 |
| Age | | 0.027 | 0.072 | 0.377 | .701 | | 0.041 | 0.075 | 0.543 | .588 |
| Overcommitment | | 0.047 | 0.086 | 0.541 | .590 | | -0.064 | 0.090 | -0.705 | .482 |
| Anxiety | | -0.019 | 0.090 | 0.210 | .834 | | -0.098 | 0.091 | -1.078 | .283 |
| Fatigue | | 0.063 | 0.086 | 0.732 | .465 | | -0.057 | 0.090 | -0.643 | .949 |
| LF/HF baseline | | -0.023 | 0.099 | -0.228 | .820 | | -0.032 | 0.103 | -0.310 | .757 |
| ERI ratio x LF/HF | | 0.063 | 0.093 | 0.680 | .497 | | 0.139 | 0.096 | 1.441 | .152 |
| ERI ratio x Gender | | -0.058 | 0.078 | 0.745 | .445 | | -0.113 | 0.081 | -1.396 | .164 |
| ERI ratio x LF/HF x Gender | | 0.042 | 0.095 | 0.438 | .662 | | 0.079 | 0.098 | 0.797 | .427 |

*Note*. DET-VR total model *R^2^*= .04, *F*_11,139_ = 0.432, *p* = .948; IDN-VR total model, *R^2^*= .11, *F*_11,136_ = 1.380*, p* =.183; Gender; 1= male, 2 = female. All covariates that define products were mean centred within the PROCESS program. Multivariate outliers (DET-VR, n = 11, IDN-VR, n = 14) were removed based on standardized residual, leverage, and covariance ratio scores for each regression.

Supplementary Table 2. Assessing the direct and interactive effects of academic stress (ERI ratio), gender, and heart rate variability on performance on saccadic reaction time (SAC-VR).

|  | SAC-VR | | | |
| --- | --- | --- | --- | --- |
| Predictors | *β* | *se* | *t* | *p* |
| ERI ratio (continuous) | 0.035 | 0.071 | 0.495 | .622 |
| Gender | 0.011 | 0.059 | 0.196 | .847 |
| LF/HF CONVIRT | -0.123 | 0.084 | -1.470 | .144 |
| Age | -0.002 | 0.055 | -0.043 | .966 |
| Overcommitment | -0.037 | 0.067 | -0.557 | .579 |
| Anxiety | -0.072 | 0.067 | -1.072 | .286 |
| Fatigue | 0.040 | 0.066 | 0.611 | .542 |
| LF/HF baseline | -0.023 | 0.076 | -0.306 | .760 |
| ERI ratio x LF/HF | 0.020 | 0.073 | 0.271 | .787 |
| ERI ratio x Gender | -0.020 | 0.060 | 0.293 | .770 |
| ERI ratio x LF/HF x Gender | 0.059 | 0.074 | 0.798 | .426 |

*Note.* SAC-VR total model, *R^2^* = .04, *F_11,138_* = 1.45, *p* = .150; Gender; 1= male, 2 = female. All covariates that define products were mean centred within the PROCESS program. Multivariate outliers (n = 12) were removed based on standardized residual, leverage, and covariance ratio scores.
